# Beneficial effect of omarigliptin on diabetic patients with non-alcoholic fatty liver disease/non-alcoholic steatohepatitis

**DOI:** 10.1186/s13098-021-00644-5

**Published:** 2021-03-10

**Authors:** Sachiko Hattori, Kazuomi Nomoto, Tomohiko Suzuki, Seishu Hayashi

**Affiliations:** 1 Department of Endocrinology and Metabolism, Tohto Clinic, 4-1 Kioi-cho, Chiyoda-ku, Tokyo, 102-0094 Japan; 2 Department of Internal Medicine, Tohto Clinic, Tokyo, Japan; 3 Department of Gastroenterology, Ohkubo Hospital, Tokyo, Japan

**Keywords:** NAFLD/NASH, Omarigliptin, Dipeptidyl peptidase 4

## Abstract

**Background:**

Dipeptidyl peptidase 4 (DPP4) is a serine exopeptidase able to inactivate various oligopeptides, and also a hepatokine. Hepatocyte-specific overexpression of DPP4 is associated with hepatic insulin resistance and liver steatosis.

**Method:**

We examined whether weekly DPP4 inhibitor omarigliptin (OMG) can improve liver function as well as levels of inflammation and insulin resistance in type 2 diabetic patients with non-alcoholic fatty liver disease (NAFLD). Further, we investigated the effects of OMG in a diabetic patient with biopsy-confirmed nonalcoholic steatohepatitis (NASH).

**Results:**

In NAFLD patients, OMG significantly decreased levels of aminotransferase, aspartate aminotransferase, gamma-glutamyl transpeptidase, homeostatic model assessment of insulin resistance (HOMA-IR), and high-sensitivity C-reactive protein (hsCRP), while no significant change was seen in hemoglobin A1c or body mass index. In the NASH patient, liver function improved markedly, and levels of the hepatic fibrosis marker FIB-4 decreased in parallel with HOMA-IR and hsCRP. Slight but clear improvements in intrahepatic fat deposition and fibrosis appeared to be seen on diagnostic ultrasonography.

**Conclusion:**

Weekly administration of the DPP4 inhibitor OMG in ameliorating hepatic insulin resistance may cause beneficial effects in liver with NAFLD/NASH.

## Background

Dipeptidyl peptidase 4 (DPP4) is a serine exopeptidase able to inactivate various oligopeptides through the removal of N-terminal dipeptides [1]. The activity of DPP4 seems to be increased in patients with type 2 diabetes, and various in vitro and in vivo studies have demonstrated that this enzyme can interact with proinflammatory pathways [1]. DPP4 is also a hepatokine [2], and levels of this enzyme have thus been seen to be elevated in chronic liver diseases including hepatitis C, hepatitis B, non-alcoholic fatty liver disease (NAFLD) and hepatocellular carcinoma.

A direct association exists between DPP4 activity and insulin resistance in humans [3]. Evidence suggests that obesity in mice stimulates hepatocytes to synthesize and secrete DPP4, in turn promoting inflammation of adipose tissue macrophages and insulin resistance. Interestingly, silencing expression of DPP4 on hepatocytes suppressed inflammation of visceral adipose tissue and insulin resistance, but this effect did not occur with sitagliptin, an orally administered DPP4 inhibitor [4].

We recently reported that omarigliptin (OMG), a potent, selective, DPP4 inhibitor with a half-life that allows weekly dosing, decreased inflammation and insulin resistance without affecting hemoglobin A1c (HbA1c) or body mass index (BMI) in patients with type 2 diabetes, but daily DPP4 inhibitors such as sitagliptin did not change levels of inflammation and insulin resistance [5].

Since hepatic expression of DPP4 is associated with NAFLD [6], we examined whether OMG improves liver function as well as levels of inflammation and insulin resistance in type 2 diabetic patients with NAFLD. Furthermore, we administered OMG to a diabetic patient with biopsy-confirmed nonalcoholic steatohepatitis (NASH) and examined the effects.

## Method

### Study design

Study for NAFLD: This was a post hoc investigation of a previously reported study (*UMIN Clinical Registry (UMIN000029288))*[5], which included a total of 84 patients with HbA1c > 6.0% regardless of diet, exercise, and medical treatment with the DPP4 inhibitors sitagliptin (50 mg/day) or linagliptin (5 mg/day) for ≥ 12 months in this clinic: Patients were allocated in a 1:2 ratio using numbered containers to continue with the same daily regimens of sitagliptin 50 mg/day (n = 19) or linagliptin 5 mg/day (n = 9) as a control group (n = 28) or to switch from sitagliptin (n = 40) or linagliptin (n = 16) to OMG 25 mg/week (OMG group: n = 56). For this NAFLD study, NAFLD was retrospectively diagnosed using data from ultrasonography performed at enrollment for 12 patients in the control group and 21 patients in the OMG group. In these NAFLD patients, changes from baseline to 1 year for HbA1c, BMI, homeostatic model assessment of insulin resistance (HOMA-IR), high-sensitivity C-reactive protein (hsCRP), alanine aminotransferase (ALT), aspartate aminotransferase (AST), and gamma-glutamyl transpeptidase (γGTP) were evaluated.

Study for NASH: We further investigated OMG in a patient with biopsy-confirmed NASH. This patient was a 73-year-old man who had been found to have fatty liver on abdominal ultrasonography at about 35 years old. Type 2 diabetes and dyslipidemia were then found at about 40 years old, and he started therapy at about 50 years old. He did not modify his lifestyle habits in any particular manner and continued to eat a high-fat, high-salt diet and smoke (20 cigarettes/day for 50 years), but not drink alcohol. We measured HbA1c, BMI, HOMA-IR, hsCRP, liver function, and hepatic fibrosis markers in this patient, and results of diagnostic ultrasonography were tracked over time.

### Criteria for NAFLD diagnosis

NAFLD was diagnosed by ultrasonography according to the presence of one of the following criteria: (i) bright homogeneous echoes in the liver parenchyma; (ii) hepatorenal echogenicity contrast ( +); (iii) hepatosplenic echogenicity contrast ( +); (iv) echoes with deep attenuation in the liver parenchyma; or (v) impaired visualization of the peripheral portal and hepatic veins. Exclusion criteria were a history of hepatic diseases, such as hepatitis C, hepatitis B, or primary biliary cirrhosis, or a past history of alcohol consumption > 20 g/day.

### Statistical analysis

Paired t-tests were used to compare parameters before treatment and at 12 months after treatment. Differences were considered statistically significant at the level of p < 0.05.

## Results

### Effect of OMG in diabetic patients with NAFLD

No significant differences were seen in any parameters in the control group. In the OMG group, significant differences were observed in ALT, AST, γGTP, HOMA-IR, and hsCRP, while no significant differences were seen in HbA1c or BMI (Table 1).

**Table 1:** Parameters in control and omarigliptin group with NAFLD at baseline and 12 months

Time (month)	Control (n = 12)		Omarigliptin (n = 21)	
	0	12	0	12
HbA1c	6.83 ± 0.69	6.80 ± 0.63	6.91 ± 0.73	6.80 ± 0.71
BMI	26.5 ± 2.4	26.7 ± 2.1	26.6 ± 2.7	26.5 ± 2.7
ALT	30.8 ± 19.5	32.4 ± 28.4	30.1 ± 20.5	25.2 ± 14.4 *
AST	35.1 ± 8.4	37.5 ± 10.0	34.8 ± 11.6	30.0 ± 8.6 *
γGTP	38.5 ± 6.6	39.0 ± 5.5	43.8 ± 29.6	32.7 ± 19.8 *
hsCRP	0.130 ± 0.04	0.151 ± 0.06	0.105 ± 0.05	0.042 ± 0.02 **
HOMA-IR	2.83 ± 0.79	2.72 ± 0.82	2.75 ± 1.28	1.85 ± 0.88 **

Data were expressed as mean ± standard deviation. (*p < 0.05, **p < 0.01)

### Effect of OMG in a diabetic patient with NASH

**Fig. 1:**
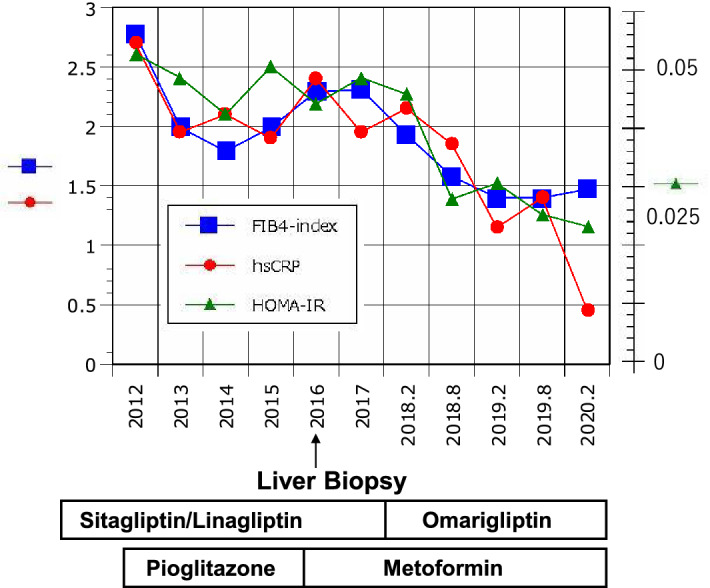
Time course of FIB-4 index, HOMA-IR, and hsCRP in a type 2 diabetic patient with NASH. FIB-4: blue squares; HOMA-IR: green triangles; hsCRP: red circles. FIB-4 = age ([yr] × AST [U/L]) / ((PLT [10^9^/L]) × (ALT [U/L])(1/2))

This patient was referred to our department at 64 years old for worsened liver function and poor glycemic control. The results of physical examination at the initial consultation were as follows: height, 182 cm; weight, 74.1 kg; BMI, 22.37 kg/m^2^; blood pressure, 128/80 mmHg; heart rate, 78 beats/min and regular; no anemia or jaundice; electrocardiography and chest X-ray, no findings of note; abdominal examination, no subjective symptoms; bilateral patellar and Achilles tendon reflexes, normal; diabetic retinopathy and neuropathy, absent; and diabetic nephropathy stage I (albumin/creatinine ratio, 5.6 mg/g creatinine). After the initial treatment with glimepiride 2 mg/day and sitagliptin 100 mg/day, laboratory results were: AST, 69 IU/L; ALT, 83 IU/L; fibrosis-4 (FIB-4) index [7], 2.78; Mac-2-binding protein glycosylation isomer (M2BPGi), 1.12; HbA1c, 7.8%; HOMA-IR, 2.61; and hsCRP, 0.054 mg/dL. Pioglitazone was then prescribed at 15 mg/day, with the dose subsequently increased to 30 mg/day. Moreover, after switching from sitagliptin to linagliptin, laboratory results improved as follows: AST, 45 IU/L; ALT, 52 IU/L; HbA1c, 7.2%; and HOMA-IR, 2.1. At 68 years old, laboratory results again worsened: AST, 61 IU/L; ALT, 79 IU/L; FIB-4 index, 2.29; M2BPGi, 1.14; HbA1c, 7.4%, HOMA-IR, 2.19; and hsCRP, 0.048 mg/dL. In response, pioglitazone was switched to metformin 1000 mg/day, which led to an improving trend, with HbA1c at 6.9%, but no changes in liver function or hepatic fibrosis markers. Liver biopsy was then performed, and NASH (Brunt criteria: grade 1, stage 3) was diagnosed, indicating better control of diabetes mellitus as a critical issue. Therapy was switched from linagliptin to OMG, which has wide organ distribution including the liver, is present stably in the body without accumulation, and is safe to use [8]. Twenty-four months later, liver function had improved markedly: AST, 20 IU/L; ALT, 19 IU/L; FIB-4, 1.47; M2BPGi, 0.58; HbA1c, 6.4%; HOMA-IR, 1.26; and hsCRP, 0.028 mg/dL (Table 2). The hepatic fibrosis marker FIB-4 changed in parallel with HOMA-IR and hsCRP (Fig. 1). Thereafter, slight but clear improvements in intrahepatic fat deposition and fibrosis were seen on diagnostic ultrasound imaging systems.

**Table 2:** Time course of clinical parameters in a patient with NASH

Date	2012	2013	2014	2015	2016	2017	2018	2019	2019	2020	2020
BMI	22.3	22.4	22.3	22.1	22.1	22.2	22.3	22.4	22.4	22.3	22.3
AST (U/L)	69	48	45	48	61	54	47	28	23	20	21
ALT (U/L)	83	60	52	60	79	55	55	33	26	19	19
Plt (10(9)/L)	198	212	227	223	204	214	227	218	228	233	243
FIB-4	2.78	1.99	1.79	1.99	2.29	2.31	1.93	1.58	1.4	1.4	1.47
Mac2BPGi	1.12	0.93	0.99	1.01	1.14	1.03	1.09	0.83	0.72	0.68	0.58
Type4Collagen					209	191	211	186	179	174	165
(ng/ml)											
hsCRP (mg/dl)	0.054	0.039	0.042	0.038	0.048	0.039	0.04	0.04	0.023	0.028	0.009
FBS (mg/dl)	158	148	149	163	178	160	156	128	134	114	124
HbA1c (%)	7.8	6.8	7.2	7.6	7.4	6.9	6.6	6.5	6.5	6.4	6.5
IRI (IU/ml)	6.7	6.6	5.7	6.2	5	6.1	5.9	4.4	4.6	4.5	3.8
HOMA-IR	2.61	2.41	2.1	2.5	2.19	2.41	2.27	1.39	1.52	1.26	1.16
TG (mg/dl)	116	155	133	116	132	150	153	73	75	126	89
Total-C (mg/dl)	175	173	193	175	179	151	157	152	141	171	158
HDL-C (mg/dl)	37	34	43	37	41	36	358	41	41	45	43
LDL-C (mg/dl)	115	108	120	115	110	85	88	96	85	101	98
rosuvastatin (mg/day)	2.5	2.5	2.5	2.5	2.5	2.5	2.5	2.5	2.5	2.5	2.5
glimepirid (mg/day)	2	2	2	2	2	2	2	2	2	2	2
sitagliptin (mg/day)	100										
linagliptn (mg/day)	5	5	5	5	5	5					
omarigliptin (mg/week)							25	25	25	25	25
pioglitazone (mg/day)	15	30	30	30							
metoformin (mg/day)					1000	1000	1000	1000	1000	1000	1000
					**↑ **Liver Biopsy						

FIB-4 = age ([yr] x AST [U/L]) / ((PLT [10(9)/L]) x (ALT [U/L])(1/2))

M2BPGi: Mac-2 binding protein glycosylation isomer

## Discussion

NAFLD is primary characterized by the accumulation of intrahepatic triglycerides (TGs) and is present in 75–90% of subjects with type 2 diabetes [9, 10]. NAFLD may progress to the more severe condition of NASH, characterized by advanced histological remodeling including fibrosis, lobular inflammation, hepatocellular ballooning, and risk of liver cancer. Since numerous pathways including insulin resistance, lipotoxicity, oxidative stress, immunology, the cytokine system, mitochondrial damage, and apoptosis are involved in the pathophysiology of NASH, various pharmacotherapies are being developed. Although no presently available drugs can be recommended for evidence-based treatment of NASH, antidiabetic drugs may prove useful in patients with comorbid diabetes mellitus.

We found that change from daily DPP4 inhibitors to OMG appears to offer benefits for NAFLD patients along with decreased insulin resistance and inflammation. Based on this experience, we tried OMG by changing from linagliptin on a NASH patient in whom glycemic control, liver function, and hepatic fibrosis markers improved markedly, along with decreased HOMA-IR and hsCRP, and slight but clear improvements in intrahepatic fat deposition and fibrosis were seen on diagnostic ultrasound imaging systems.

DPP4 has been linked to hepatic insulin sensitivity in several studies. Thus, in mice, hepatocyte-specific overexpression of DPP4 is associated with hepatic insulin resistance and liver steatosis [6], whereas knockdown of DPP4 improves insulin sensitivity and reduces lipid accumulation in cultured hepatocytes [11]. Other studies have pointed toward DPP4 acting as a hepatokine, linking the liver and adipose tissue with the development of insulin resistance, and glucose intolerance. In mice, obesity and the associated visceral adipose tissue inflammation result in insulin resistance, a process that appears to be mediated via increased synthesis and release of hepatic DPP4, since eliminating hepatocyte DPP4 expression suppresses inflammation and improves insulin sensitivity [4, 12]. The mechanism seems independent of the catalytic activity of DPP4, since these effects were not mimicked by systemic daily DPP4 inhibition [4, 12]. On the other hand, inhibition of the catalytic activity of DPP4 using DPP4 inhibitors was suggested to be, at least partially, involved since insulin signaling was improved following inclusion of DPP4 inhibitors in adipocytes in culture, but the mechanism remained unidentified [13, 14]. Although the mechanisms how changing from daily DPP4 inhibitors to weekly OMG causes beneficial effect in liver with NAFLD/NASH are unclear, decreasing effect of OMG on inflammation and insulin resistance probably in liver might be involved.

DPP4 is proposed to represent a novel adipokine that may impair insulin sensitivity in autocrine and paracrine fashions [13]. DPP4 release strongly correlates with adipocyte size, potentially representing an important source of DPP4 [13]. The greater the fat content in the liver, the greater the expression/secretion of hepatokine DPP4, which might lead to NAFLD, and then to NASH in autocrine and paracrine fashions. OMG might thus block the activity of DPP4 highly secreted from the liver under conditions of NAFLD/NASH, probably averting the promotion of adipose inflammation and insulin resistance in liver.

Accordingly, excess DPP4 derived from adipocytes and/or hepatocytes may act as a local mediator of inflammation and adipose/hepatic tissue insulin resistance, thereby forming a link between obesity and the pathogenesis of type 2 diabetes and metabolic disease. Sodium-glucose transporter 2 inhibitors and glucagon-like peptide 1 receptor agonists have recently shown potential efficacy for the treatment of NAFLD/NASH with diabetes [1, 15, 16], but are expected to be more effective for NAFLD/NASH in obese diabetic patients. The possible effects of OMG in decreasing intrahepatic fat accumulation and improving intrahepatic adipose inflammation may be helpful for the treatment of NAFLD/NASH, particularly in non-obese, insulin-resistant, diabetic patients like a NASH case described here.

The principal limitation of the present study was the relatively small number of participants. Since this is a novel possible therapeutic for NAFLD/NASH in patients complicated with diabetes, long-term assessments in a larger number of patients are necessary.

## Conclusion

Hepatocyte-specific overexpression of DPP4 is associated with hepatic insulin resistance and liver steatosis. Weekly administration of the DPP4 inhibitor OMG in ameliorating hepatic insulin resistance may cause beneficial effects in liver with NAFLD/NASH.

## Data Availability

The datasets analyzed during the current study are not publicly available due to some relevant ongoing studies, but may be available from the corresponding author of this article on reasonable request.

## Abbreviations

ALT: Alanine aminotransferase
AST: Aspartate aminotransferase
BMI: Body mass index
DPP4: Dipeptidyl peptidase 4
FIB-4 index: Fibrosis-4 index
GLP-1: Glucagon-like peptide-1
FBG: Fasting blood glucose
HbA1c: Hemoglobin A1c
HDL: High-density lipoprotein
HOMA-IR: Homeostatic model assessment of insulin resistance
hsCRP: High-sensitivity C-reactive protein
M2BPGi: Mac-2-binding protein glycosylation isomer
NAFLD: Non-alcoholic fatty liver disease
NASH: Non-alcoholic steatohepatitis
OMG: Omarigliptin
TG: Triglyceride
γGTP: Gamma-glutamyl transpeptidase

## References

[1] Godoy-Matos AF, Silva Júnior WS, Valerio CM (2020). NAFLD as a continuum: from obesity to metabolic syndrome and diabetes. Diabetol Metab Syndr..

[2] Silva Júnior WS, Souza MDGC, Kraemer-Aguiar LG (2018). Dipeptidyl peptidase 4 (DPP4), adipose inflammation, and insulin resistance: is it time to look to the hepatocyte?. Hepatobiliary Surg Nutr.

[3] Silva Júnior WS, Souza MDGC, Nogueira Neto JF (2019). Dipeptidyl peptidase 4 activity is related to body composition, measures of adiposity, and insulin resistance in subjects with excessive adiposity and different degrees of glucose tolerance. J Diabetes Res.

[4] Ghorpade DS, Ozcan L, Zheng Z (2018). Hepatocyte-secreted DPP4 in obesity promotes adipose inflammation and insulin resistance. Nature.

[5] Hattori S (2020). Omarigliptin decreases inflammation and insulin resistance in a pleiotropic manner in patients with type 2 diabetes. Diabetol Metab Syndr..

[6] Baumeier C, Schlüter L, Saussenthaler S (2017). Elevated hepatic DPP4 activity promotes insulin resistance and non-alcoholic fatty liver disease. Mol Metab.

[7] Sterling RK, Lissen E, Clumeck N (2006). Development of a simple noninvasive index to predict significant fibrosis in patients with HIV/HCV coinfection. Hepatology.

[8] Omarigliptin Pharmaceutical interview form 2016; 4th edition.

[9] Klöting N, Fasshauer M, Dietrich A (2010). Insulin-sensitive obesity. Am J Physiol Endocrinol Metab.

[10] Younossi ZM, Koenig AB, Abdelatif D (2016). Global epidemiology of nonalcoholic fatty liver disease-Meta-analytic assessment of prevalence, incidence, and outcomes. Hepatology.

[11] Rufinatscha K, Radlinger B, Dobner J (2017). Dipeptidyl peptidase-4 impairs insulin signaling and promotes lipid accumulation in hepatocytes. Biochem Biophys Res Commun.

[12] Varin EM, Mulvihill EE, Beaudry JL (2018). Circulating levels of soluble dipeptidyl peptidase-4 are dissociated from inflammation and induced by enzymatic DPP4 inhibition. Cell Metab.

[13] Lamers D, Famulla S, Wronkowitz N (2011). Dipeptidyl peptidase 4 is a novel adipokine potentially linking obesity to the metabolic syndrome. Diabetes.

[14] Röhrborn D, Brückner J, Sell H, Eckel J (2016). Reduced DPP4 activity improves insulin signaling in primary human adipocytes. Biochem Biophys Res Commun..

[15] Akuta N, Watanabe C, Kawamura Y (2017). Effect of sodium-glucose cotransporter 2 inhibitor in nonalcoholic fatty liver disease complicated by diabetes mellitus: preliminary prospective study based on serial liver biopsies. Hepatol Commun.

[16] Dong Y, Lv Q, Li S (2017). Efficacy and safety of glucagon-like peptide-1 receptor agonists in non-alcoholic fatty liver disease: a systemic review and meta-analysis. Clin Res Hepatol Gastroenterol.

